# Global and unbiased detection of splice junctions from RNA-seq data

**DOI:** 10.1186/gb-2010-11-3-r34

**Published:** 2010-03-17

**Authors:** Adam Ameur, Anna Wetterbom, Lars Feuk, Ulf Gyllensten

**Affiliations:** 1 Department of Genetics and Pathology, Rudbeck laboratory, Uppsala University, SE-751 85 Uppsala, Sweden

## Abstract

We have developed a new strategy for *de novo *prediction of splice junctions in short-read RNA-seq data, suitable for detection of novel splicing events and chimeric transcripts. When tested on mouse RNA-seq data, >31,000 splice events were predicted, of which 88% bridged between two regions separated by ≤100 kb, and 74% connected two exons of the same RefSeq gene. Our method also reports genomic rearrangements such as insertions and deletions.

## Abstract 2

SplitSeek can be used to detect novel splicing events in SOLiD RNA-seq data without the need for a pre-defined library.

## Introduction

High-throughput sequencing of mRNA opens unprecedented opportunities to identify the spectrum of splice events in a sample on a global scale. The typical approach for detecting splicing in RNA-seq experiments has been to map the reads to a junction library consisting of predefined exon-exon boundaries [1-6]. Although these strategies can successfully recover many splice events, they do not analyze splicing from a truly global and unprejudiced perspective. Only splice junctions present in the library can be identified, and it is simply not feasible to match against all possible combinations of exons. For example, a genome with 100,000 (10^5^) exons, which is a low estimate for mammalian genomes, would yield 10^10 ^combinations. To address this problem, the size of the junction library must be reduced dramatically, and consequently, most methods consider only the candidates involving known exons within the same gene. A severe limitation with this approach is that splicing events involving previously unknown exons cannot be identified. Also, this type of analysis is restricted to the relatively small number of species in which coordinates of genes and exons have been found.

To overcome some of these limitations, the splice-junction library can instead be created directly from the RNA-seq data without relying on any genome annotations. This approach is taken by the two packages G-Mo.R-Se [7] and TopHat [8]. With these methods, all reads are first mapped to the reference genome, and transcribed fragments are identified through analysis of the coverage profile. The ends of these fragments are then combined into a library of putative exon boundaries to which the previously unmapped reads are aligned. Although this strategy has some advantages over methods that construct the library from known annotations, the problem of analyzing all possible exon combinations remains. G-Mo.R-Se and TopHat solve this problem by considering only putative junctions that span between neighboring (but not necessarily adjacent) transcribed fragments and those that contain a canonic (GT/C-AG) splice site. These restrictions imply that a substantial number of true splice junctions (for example, those with long introns or noncanonic splice sites) are outside of the detection range. A further limitation is that these methods are based on accurate *de novo *identification of exon boundaries from raw RNA-seq data, which in itself is a computationally challenging task, especially for transcripts expressed at lower levels.

An important application of deep RNA sequencing is the discovery of fusion transcripts in cancer, and two consecutive methods have been proposed by Maher and colleagues [9]. Initially the authors used a combination of long reads (>200 bp) from the Roche 454 sequencer and shorter reads from the Illumina (Solexa) platform, and later they shifted to using paired-end sequencing (2 × 50 bp) [10]. Although these strategies can successfully discover fusion transcripts, they have a number of important drawbacks. First, it is both costly and labor intensive to use two different sequencing platforms, as was done in their primary study. Second, the mate-pair approach complicates the analysis, because the expected insert size must be taken into account when estimating the expected distance between two mates in the sequenced transcript. This will be particularly problematic for mates that span over several splice junctions. Also, preparation of mate-pair libraries require larger amounts of RNA than the fragment libraries used in most RNA-seq experiments. The amount of RNA can be a crucial limitation, especially when studying clinical samples.

Here we present an alternative approach to identify splice junctions. The junctions are predicted *de novo *without any preassumed set of allowed exon boundaries. This implies that all types of splicing events in the RNA sample can be detected in a completely unbiased way, including previously unknown splice junctions and fusion transcripts. Also, we rely entirely on short reads (~50 bp) from fragment libraries, which is the type of RNA-seq data normally generated by using the Illumina or SOLiD platforms. By applying our method to available RNA-seq data from mouse cells [6], we showed that splice junctions can be identified at almost nucleotide precision and with a very low false-discovery rate (FDR). Moreover, this strategy also allows unbiased detection of insertions, deletions, and other types of genomic rearrangements within transcribed sequences. Indels and coding repeat expansions are important in a large number of human disorders [11]. The potential for simultaneous detection of expression levels and coding-sequence variation in a single analysis pipeline will be beneficial for patient-sample analysis. We have implemented our method in a software called SplitSeek. The SplitSeek results can be directly uploaded to the UCSC genome browser [12] and used as input to the BEDTools software suite [13], which enables the user to visualize and analyze the predicted events in a genomic context.

## Results

**Figure 1:**
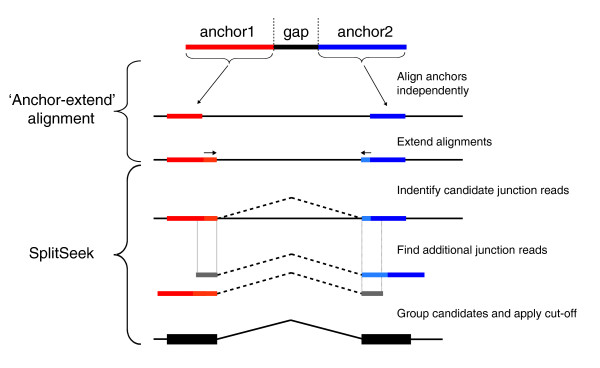
**Overview of the split-read strategy**. Each read is split into two pieces, or "anchors," of equal length (red and blue), with a gap between them. The anchors are aligned independently, and only the instances in which both align uniquely to the reference sequence are considered. Then, the alignments are extended as long as they still match the reference sequence. The SplitSeek program identifies all candidate junction reads from the split-read alignments where the boundary is located in the gap between the anchors. Then additional junction reads are detected from the set of reads that partly align to a previously detected candidate junction, and where the remaining, nonaligned, part of the read (grey lines) has a 5-bp identical sequence compared with the corresponding part of the same candidate read. SplitSeek then groups all potential junction reads, applies cut-offs, and reports the results.

Our strategy consists of a combination of a split-read alignment and the novel SplitSeek program (see Figure 1). In the alignment, every read is split into two nonoverlapping parts, or "anchors," that are aligned separately. The two anchors are then extended as long as they still match the reference sequence. If a splice junction is located in the gap between the two anchors, then the two parts are matched to different genomic positions (that is, the two exons in the junction). The SplitSeek program then performs a number of analysis steps to predict the exon boundaries. First, all instances of split reads are found, and their genomic positions and nucleotide sequence are recorded. They comprise the initial set of candidates, and all resulting splice events will be found among these. However, many reads exist in which the junction is located in one of the anchors rather than in the gap. To identify such additional junction reads, we scan all reads in which only one of the anchors was aligned. If such an anchor can be extended to the exact position as a previously identified candidate junction, and the sequence in the two reads aligns perfectly within the first five bases of the other exon (gray lines in Figure 1), then the read is considered to confirm the junction. This implies that SplitSeek can find junction reads in which as few as five bases overlap with the other exon. In the final step, all identified junction reads are grouped, and user-defined cut-offs are applied to obtain a final set of exon boundaries. Because this method is unbiased, it will report all types of events in which a read must be split to match the reference genome, including small insertions and deletions.

In this study, we evaluated our method on public RNA-seq data from single mouse oocytes [6], sequenced on the SOLiD platform. The analysis was performed on two independent samples, oocyte1 (with 11.6 million reads) and oocyte2 (23.5 million reads), and oocyte1+2, a combination of all reads from the two samples. These data consist of 50-bp reads, and the alignment was performed by using the AB/SOLiD whole-transcriptome-alignment software with anchor lengths in the range between 21 and 24 (see Methods for details). The highest number of uniquely mapped split reads was obtained for lengths 22 and 23 (see Table 1), probably because shorter splits do not align uniquely to the genome, whereas the longer do not give a sufficiently large gap. We therefore selected 22 as the anchor length in the remaining analysis.

**Table 1:** Number of split read alignments

	Oocyte 1	Oocyte 2
Anchor length 21	110468	203159

Anchor length 22	157138	284468

Anchor length 23	158487	284579

Anchor length 24	143293	257316

We required each junction to be supported by at least two uniquely positioned reads in the SplitSeek analysis, and a summary of the results is presented in Table 2. Between 17,397 and 31,532 junctions were predicted in the three samples, with 93% to 88% of them bridging between regions on the same chromosome, separated by ≤100 kb, and ≥74% mapping within five bases of a known exon-exon boundary in an RefSeq gene. The numbers suggest that our method has a very low false-positive rate, and to support this further, we estimated the false-discovery rate (FDR) for all junctions within 1 Mb and 100 kb, respectively (see Methods for details). The FDR was <1 in 1,000 for junctions within 1 Mb and <1/10,000 for those within 100 kb. Naturally, the FDR will be higher for splicing events that are farther apart than 1 Mb or on different chromosomes. However, such instances comprise a small subset of all junctions, and they can either be disregarded or be examined individually, depending on the aim of the study. Also, it is possible to increase the specificity by requiring three uniquely positioned reads or more for each predicted junction.

**Table 2:** Splice junctions and insertions reported by SplitSeek with anchor length 22

	Oocyte 1	Oocyte 2	Oocyte 1+2
Number processed reads	11,565,660	23,488,851	35,054,511

Predicted splice junctions	17,397	23,703	31,532

Within chromosome	16,205 (93.1%)	21,495 (90.7%)	27,957 (88.7%)

Within 1 Mb	16,128 (92.7%)	21,374 (90.2%)	27,757 (88.0%)

Within 100 kb	16,094 (92.5%)	21,323 (90.0%)	27,685 (87.8%)

Match to a RefSeq exon-exon boundary^a^	14,264 (82.0%)	18,139 (76.5%)	23,235 (73.7%)

Expected false within 1 Mb (FDR)	12.9 (8.0·10^-4^)	17.6 (8.2·10^-4^)	23.4 (8.4·10^-4^)

Expected false within 100 kb (FDR)	1.3 (8.0·10^-5^)	1.8 (8.2·10^-5^)	2.3 (8.4·10^-5^)

Predicted insertions	275	553	834

^a^Each of the exon boundaries located within 5 bp of predicted junction.

**Figure 2:**
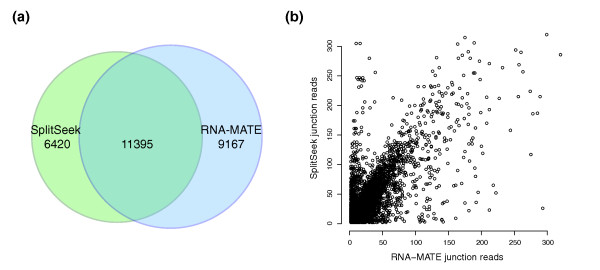
**Comparison of predictions from RNA-MATE and SplitSeek**. **(a)** Venn diagram showing the number of predicted junctions by the two methods. **(b)** Predicted number of junction reads for all for all 11,395 exon boundaries reported by both RNA-MATE (x-axis) and SplitSeek (y-axis).

The SplitSeek predictions show high specificity, but we were also interested to evaluate the sensitivity. Therefore, we compared the SplitSeek results with RNA-MATE [5], a method that recursively maps reads to a junction library of known exons. By applying the RNA-MATE program to the oocyte1 dataset (see Methods for details), we found 20,562 exon boundaries supported by at least two reads, slightly more than the 17,397 junctions predicted by SplitSeek (see Table 2). As shown in Figure 2a, 11,395 splice junctions were detected in common, meaning that SplitSeek confirms 55% of the RNA-MATE predictions. There could be several possible reasons that the remaining 45% are not detected by SplitSeek and we believe it is due to a combination of (a) junctions at which no read is centered over the boundary and thereby is undetectable by SplitSeek; (b) junctions uniquely mappable when using an exon-junction library but not with the anchor-extend alignment; and (c) junctions falsely detected by RNA-MATE. Of the SplitSeek boundaries, 6,420 were not found by RNA-MATE, and 1,007 (16%) of these were long-range splicings of ≥100 kb, a number that could be indicative of the false-positive rate among the junctions predicted only by SplitSeek. Interestingly, as many as 4,069 (63%) of the 6,420 SplitSeek-only predictions coincide with RefSeq exon boundaries. These can be explained partly by the fact that the RNA-MATE library was not completely up to date (see Methods), but as many as 2,519 of these junctions were present in the library file, which demonstrates that a substantial number of splice events are detectable only by SplitSeek. However, a large number of exon boundaries were reported by both methods, and for these, we could see a clear correlation in the number of reads predicted to cover the junctions (see Figure 2b). The scatterplot shows a systematic bias toward more reads/junction for SplitSeek, probably because SplitSeek can use reads in which only five nucleotides are sequenced from the other exon, whereas this overhang must be longer for library-based methods. A peculiar observation is a group of points in the upper left corner, with many reads for SplitSeek and few for RNA-MATE. We think that these largely represent cases in which RNA-MATE predicts two or more highly similar splice events located only a few bases apart, whereas SplitSeek groups them into one single junction. In such cases, the RNA-MATE junctions, each with varying number of reads, will be compared with one single SplitSeek prediction based on all junction reads, and consequently, some of the points might end in the top-left corner of Figure 2b. However, it remains unclear whether these highly similar junctions reflect real splicing events or if they are artifacts from the library construction and mapping procedures. In conclusion, this comparison suggests that junction library-based methods and SplitSeek can complement each other to detect more splice variants in known genes.

**Figure 3:**
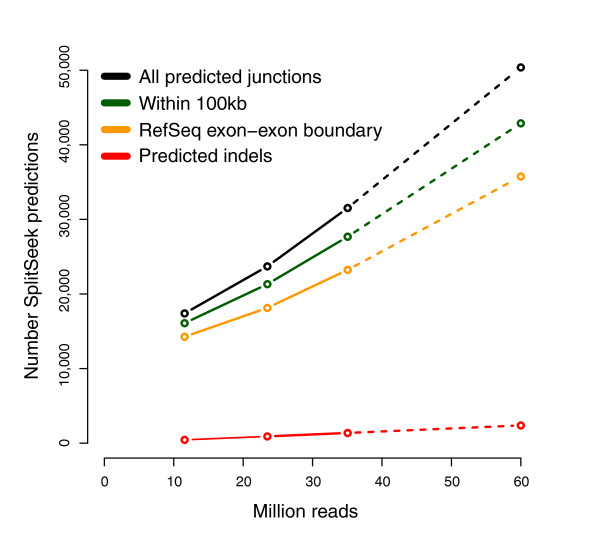
**Number of predicted splice junctions (y-axis) as a function of the total number of processed reads (x-axis)**. The number of predicted junctions (black line) increases almost linearly with the number of reads. The green and orange lines represent two subgroups of predicted junctions: those where the two boundaries are separated by ≤100 kb, and those connecting two exon boundaries of a RefSeq gene. Predicted insertions and deletions are combined and represented by the red line.

**Figure 4:**
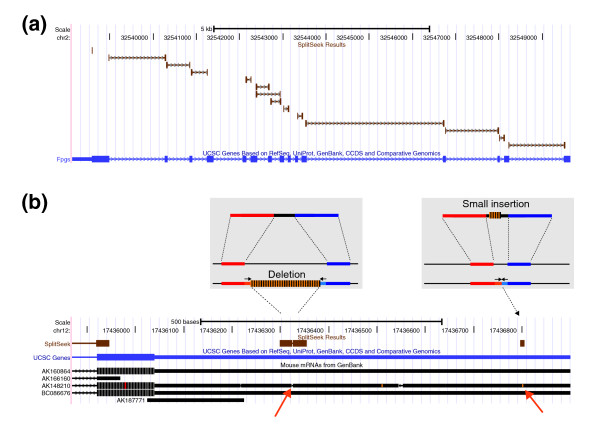
**SplitSeek results viewed in the UCSC genome browser**. **(a) **Predicted splice junctions in the gene *Fpgs*. **(b) **The two grey boxes give a schematic view of how deletions and insertions are detected. The genome browser image below shows the SplitSeek results in the last exon and 3' UTR of the *Nol10 *gene on chromosome 12. Three events are predicted, a splice junction (to the left), a deletion (in the middle,) and an insertion (to the right). The predicted insertion and deletion are both supported by the mRNA AK148210, as indicated by the orange arrows at the bottom.

As seen in Figure 3, an almost a linear correlation exists between the number of SplitSeek predictions and the total number of reads in the three samples. This demonstrates that we have not yet reached saturation and would detect many more splice junctions by deeper sequencing, as indicated by extrapolated dotted lines in Figure 3. The SplitSeek results can be viewed in the UCSC genome browser [14], as illustrated by two example regions in Figure 4. The first example shows a gene with many predicted exon-exon boundaries, including alternative splicing (Figure 4a), whereas the second demonstrates the possibility of detecting insertions/deletions in the sample (Figure 4b). In both cases, the SplitSeek predictions agree with annotated splice junctions, insertions, and deletions almost at nucleotide resolution. The reason that the position is not always exact is that the first few nucleotides in an intron may coincide with the first bases of the next exon, thereby resulting in a slight overextension of the anchor during the alignment procedure.

As mentioned earlier, a special feature of our split-read strategy is that it also can find indels (see Figure 4b). In these oocyte RNA samples, SplitSeek predicted 834 small insertions of up to six nucleotides, supported by at least two unique reads (Table 2), and 647 of these were found inside RefSeq exons. More specifically, 502 (78%) of these 647 insertions are located in the 3'UTR (see Table 3), where a higher degree of genetic variation is expected compared with the coding regions, because such events do not affect the amino acid sequence of the translated protein. By comparison, the combined lengths of 3'UTRs make up 46% of the total length of RefSeq exons, indicating a selective constraint against small insertions in coding sequence compared to untranslated regions. Deletions are somewhat more complicated to identify since they appear identical to splice junctions. Here we considered only the cases in which the two alignments are located within the same exon to represent a putative deletion, because it is unlikely that this would correspond to a true splicing event. In this manner, we predicted 536 deletions, with 343 (64%) located in the 3'UTRs (Table 3). The lower percentage of deletions in 3'UTRs compared with insertions could be due to a small proportion of splice events being reported as deletions. SplitSeek can also output other types of rearrangements, including inversions and translocations, although such events will typically not be found in RNA-seq data.

**Table 3:** Number of predicted small insertions and deletions within RefSeq exons and 3'UTRs

	Oocyte 1	Oocyte 2	Oocyte 1+2
Insertions in RefSeq exons	222	412	647

Insertions in 3' UTR	174 (78.4%)	320 (77.7%)	502 (77.6%)

Deletions in RefSeq exons	169	355	536

Deletions in 3' UTR	113 (66.9%)	229 (64.5%)	343 (64.0%)

Deletions are required to be >5 bp from any RefSeq exon boundary.

**Figure 5:**
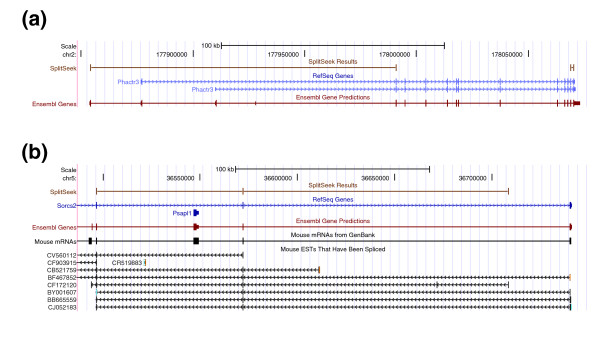
**Two long-range SplitSeek predictions (>100 kb) that extend known gene models**. **(a) **A predicted junction that connects an exon in the Ensembl Gene Prediction database with the second exon of the *Phactr3 *gene, suggesting the presence of an alternative transcription start site. **(b) **A putative novel exon in the *Sorcs2 *gene that is currently only supported by EST data.

In the SplitSeek results, ~12% of the junctions bridged between regions separated by ≥100 kb, and 26% did not connect two RefSeq exon boundaries (see Table 2). In many studies, these types of predictions might be the ones of highest interest because they could reveal novel and unexpected splicing. Up until now, it has been difficult (if at all possible) to study such events on a global scale, and therefore, we screened the SplitSeek results to see whether we could find any example of novel and long-range splicing. Interestingly, several of these predictions have strong evidence. Figure 5 shows two examples of long-range junctions (>100 kb) that bridge between RefSeq exons and regions that were previously annotated by gene prediction and EST data. Both examples in Figure 5 strongly suggest that an exon is missing in the current RefSeq annotations. This demonstrates that SplitSeek can detect novel splice events and be used as a way to extend known gene models.

## Discussion

Our results demonstrate that SplitSeek has a high specificity, and the number of false positives could be reduced even further by requiring more unique reads to cover each junction. A more difficult task is to increase the sensitivity, but our comparison with the RNA-MATE program [5] suggests that one possible way is to use SplitSeek in combination with a complementary method that aligns the reads to a library of known exon boundaries. However, this comparison is focused only on splicing between annotated exons, whereas one of the strengths of SplitSeek is that it can perform other types of analysis in which RNA-MATE or other available tools cannot be directly applied. These include identification of splice sites in uncharacterized transcripts, detection of long-range fusion transcripts, and detection of small indels in transcribed sequences.

About 12% of the predicted junctions bridge between regions separated by ≥100 kb (see Table 2). Although a few of them can probably be explained by long introns (for example, Figure 5), this can not account for all detected long-range splicing and especially not the junctions bridging between different chromosomes. Instead, it is likely that many of them are false positives because of alignment issues or properties of the genome sequence. As an example, we may falsely detect splicing between different genes that belong to the same family just because of high sequence similarity in the exons. However, we cannot rule out that a substantial number of these unexpected splicing events are indeed true, and these would be interesting to investigate further. In that case, it might be reasonable to consider only the events bridging between regions identified as significantly transcribed from the RNA-seq data to filter out a large part of the false-positive long-range splicings.

The main limiting factor in the SplitSeek method is that there must be at least one read almost centered over an exon boundary; otherwise, it will not be detectable. When using 50-bp reads and 22-bp anchors as in this study, seven (14%) of 50 of the junction reads have this property. With a length of 75 bp and still splitting into 2 × 22 bp, this proportion would increase to 32 (43%) of 75, and this would likely increase the number of detected splicing events significantly. Another benefit of longer reads is that they could allow longer anchor lengths in the alignment, which might be necessary to discover junctions that are not uniquely mappable with shorter reads. However, it also is possible to increase the throughput by simply performing a deeper sequencing by using more of the 50-bp reads, and it is not obvious which is the optimal approach for this application. Although several benefits exist of using longer reads, some drawbacks might also occur, such as lower-quality base calls at the ends of the reads and difficulties in identifying splicing between very short exons.

Because of the recent improvements in throughput of the next-generation sequencing platforms, we believe that this strategy will make it feasible to investigate the entire spectrum of splicing events or gene fusions in an RNA sample in a completely unbiased way. We also want to emphasize the possibility of finding insertions, deletions, and other types of genetic rearrangements with the SplitSeek approach. This moves beyond the scope of RNA-seq data analysis, because it can equally well be used for DNA samples sequenced with high coverage.

## Conclusions

We have developed a strategy for *de novo *detection of splice junctions in RNA-seq data. The exon-exon boundaries are identified almost at nucleotide resolution and with a low false-positive rate, <1 in 10,000 for junctions within 100 kb. Our method makes it possible to study splice junctions and fusion genes while also quantifying the gene expression, all from the same RNA-seq data. In addition, our method reports insertions and deletions in coding and noncoding parts of transcripts. We expect this to be an important application in a wide range of RNA-seq projects.

## Materials and methods

### Data acquisition and alignment

The raw RNA-seq data on mouse oocytes were downloaded from Gene Expression Omnibus [15], with accession number GSE:14605. The reads were aligned and extended by using version 1.0 of the whole transcriptome analysis tool available from Applied Biosystems [16]. This software splits each read into two parts, or "anchors," which are aligned separately and extended as far as possible while still matching the reference sequence. We matched the reads by splitting into two parts of lengths 21 to 24, allowing up to two "color space" mismatches in each alignment. The minimum score required for an alignment to be reported in the final output was set to 20.

### The SplitSeek program

Splice junctions were predicted from the alignment output files by using the SplitSeek software, which consists of two programs that are executed sequentially. In the first step, all candidate junction reads are identified and written to an intermediate BEDPE file. BEDPE is a file format that was recently introduced to give a concise description of paired-end sequence alignments [13]. This intermediate file is then used as input to a second script that performs the remaining analysis. The algorithm is split into two parts because the first program is specific to the next-generation sequencing platform, in this case, SOLiD, whereas the second script is more general.

SplitSeek finds exon-exon boundaries that are supported by several split reads. In this case, we required each junction to be covered by at least two reads with unique starting points. Other parameters that may be specified by the user include the total number of reads required to cover a predicted junction, and the maximum allowed distance between two candidate junction reads that belong to the same predicted splice junction. SplitSeek groups candidate junction reads by traversing them in the order of their genomic coordinates and joining those where the two exon boundaries are both within the allowed distance. All groups in which the number of reads is greater than the user-defined threshold are then reported in the SplitSeek output. In some cases, SplitSeek may require an additional "chrmap" input file to ensure that the chromosome names of SplitSeek predictions agree with those in the genome databases. The user is allowed to specify an upper limit on the distance between the junctions (for example, 100 kb), so that longer splicing events are not reported.

The SplitSeek results are presented in two different formats, as a BED file and a BEDPE file. The BED file can be uploaded and viewed in the UCSC genome browser, whereas the BEDPE file can be used as input to BEDTools [13] or other analysis software for comparing genomic features. SplitSeek is implemented in perl, and the program is available as Additional file 1. The code also can be downloaded from the SOLiD software-development community [17]. The current version is available for data generated by the SOLiD system, but it could be adapted to Illumina or other next-generation sequencing platforms. What then would be required is to perform a split read alignment and to write all candidate junction reads into a BEDPE formatted file to be processed by SplitSeek.

### Calculating False Discovery Rate

To make an estimate of the false discovery rate (FDR) in our results, we assume a null hypothesis in which the two parts of a splice event are uniformly distributed over the genome sequence. We then estimated an FDR for all splicing events within 1 Mb by comparing the observed values with the expected. To calculate the number of expected events, we assume that the first anchor has already been randomly mapped to the genome. In that case, the second anchor must be mapped within a ± 1-Mb window surrounding the first anchor for the criteria to be fulfilled. The size of this window is 2 × 10^6 ^bases. Because the mouse reference sequence (mm9) used in the alignment consists of about 2.7 × 10^9 ^bases, the probability that two randomly placed splicing boundaries are located within 1 Mb is ~2 × 10^6^/2.7 × 10^9 ^≈ 7.4 × 10^-4^. Under the null hypothesis, the number of expected splicing events within 1 Mb can therefore be estimated by N × 7.4 × 10^-4^, where N is the total number of predicted junctions. The FDR is then calculated as the ratio between expected/observed events. In the same way, we calculated the FDR for results within 100 kb. The results are presented in Table 2.

### Comparing SplitSeek to RNA-MATE

Version 1.01 of the RNA-MATE program was downloaded from the SOLiD software-development web page [18], along with junction library files constructed from all known genes, gene predictions, mRNA evidence, and EST evidence available at the time of creation (early 2007). The library files contains ~430,000 putative junctions, each of length 60 bp. The RNA-MATE program was then executed on the same set of reads from the oocyte1 dataset, as was used for SplitSeek. Matching in RNA-MATE was done recursively with 50-bp and 45-bp tag lengths using three allowed mismatches and default settings for all other parameters. The RNA-seq data in this experiment is not strand specific, and therefore, all junction reads from both strands were combined in the RNA-MATE output. All RNA-MATE exon boundaries with at least two reads were considered positive. A positive RNA-MATE junction was considered to coincide with a SplitSeek prediction if the difference was at most 5 bp at both ends of the junction.

## Abbreviations

EST: expressed sequence tag; FDR: false discovery rate; RNA-seq: high-throughput sequencing of RNA; 3' UTR: three prime untranslated region.

## Authors' contributions

AA and UG designed the research; AA implemented the software and conducted the analysis; and AA, AW, LF, and UG interpreted the results and wrote the manuscript.

## Supplementary Material

Additional file 1 **SplitSeek**. The SplitSeek program code, released as free software under version 3 of the GNU General Public License [19]. Click here for file
