# Supplementary material for: Global and unbiased detection of splice junctions from RNA-seq data
Source: Genome Biol. 2010 Mar 17;11(3):R34. doi: 10.1186/gb-2010-11-3-r34 (PMC2864574; doi:10.1186/gb-2010-11-3-r34)
Supplement: Additional file 1 — SplitSeek. The SplitSeek program code, released as free software under version 3 of the GNU General Public License [19]. [file gb-2010-11-3-r34-S1.gz › splitseek_v1.3.0_GB/SplitSeek_manual.pdf]

SplitSeek user manual

Version 1.3.0

License 2
Description 3
Recommendations 3
Installation 3
Split read alignment 3
SplitSeek preprocessing 4
SplitSeek 4
  Example chrmap file 5
  Output files 5
    BEDPE file 5
    BED file 6
Visualization and further analysis 6
  BEDTools 6
  UCSC genome browser 6

Adam Ameur
adam.ameur@genpat.uu.se
Department of Genetics and Pathology
Rudbeck Laboratory
Uppsala University, Sweden

License

Copyright © 2009, 2010 Adam Ameur

This program is free software: you can redistribute it and/or modify it under the terms of the GNU General Public License as published by the Free Software Foundation, either version 3 of the License, or (at your option) any later version.

This program is distributed in the hope that it will be useful, but WITHOUT ANY WARRANTY; without even the implied warranty of MERCHANTABILITY or FITNESS FOR A PARTICULAR PURPOSE. See the GNU General Public License for more details.

Description

SplitSeek is a program for *de novo* prediction of splice junctions in RNA-seq data, which can also be used for other purposes such as identification of small insertions and deletions. This user manual describes how to install and run the program.

The analysis is divided into several steps:

- **Split read alignment** - Each read is split into two non-overlapping parts that are aligned independently.
- **SplitSeek preprocessing** - Identification of candidate junction reads from split read alignments
- **SplitSeek** – Applying cut-offs and computing the final set of splice junctions and indels

Recommendations

Some important things to keep in mind before starting the analysis:

- The current SplitSeek version supports only SOLiD data
- Before running SplitSeek, the raw data must be aligned using the SOLiD Whole Transcriptome analysis pipeline (Pre-BioScope version). See more details below.
- Minimum recommended read length is 50 bases
- The number of predicted events is highly dependent on the average read depth

Installation

Download and unpack the file splitseek_vX.Y.Z.tar.gz. If necessary change the path `/usr/bin/perl` in the headers of the files 'splitseek_SOLiD_get_splice_candidates.pl' and 'splitseek.pl'.

Split read alignment

The alignment software is not part of the SplitSeek package. The current version of SplitSeek requires the split read alignment to be performed by script 'split_read_mapper.sh' included in the Pre-Bioscope version of the SOLiD Whole Transcriptome analysis pipeline. Install and run the alignment software according to the instructions available from the project webpage (http://solidsoftwaretools.com/gf/project/transcriptome/).

To increase the chance of detecting splicing events, we recommend changing the following parameters to the values below:

```
LENGTH_OF_FIRST_PART_OF_READ     22
LENGTH_OF_LAST_PART_OF_READ      22
MIN_ALIGNMENT_SCORE_FOR_REPORTING_ALIGNMENT 20
```

Since changing these parameters may increase the memory consumption, we also recommend increasing the `MAX_MEMORY_PER_JOB_IN_BYTES` parameter. Optionally, the low quality reads may be filtered out prior to the alignment since this can reduce the execution time.

SplitSeek preprocessing

One of the alignment result files should have the suffix 'max.merged.filtered.csfasta', and this file is used as input to a preprocessing script 'splitseek_SOLiD_get_splice_candidates.pl'. Below is an example how to execute the script and a description of parameters. This analysis can take several hours for large data sets.

```
>[splitseek_dir]/splitseek_solid_get_splice_candidates.pl -i [infile] -o
[outfile] -t -a -mm –silent --help
```

Parameters	
**i**	Input file. Alignment result file with suffix 'max.merged.filtered.csfasta'
**o**	Output file. A tab delimited text file on the paired-end BED (BEDPE) format. Each line contains information about the coordinates for all candidate junction reads.
**t**	Read length (default=50)
**a**	Length of 'anchors' in split read alignment (default=22)
**mm**	Maximum number of combined mismatches in both parts of the split alignment (default=no limit)

SplitSeek

The output file from the previous step is used as input to the 'splitseek.pl' program. Below is an example how to execute the script, a description of parameters and the output file.

```
>[splitseek_dir]/splitseek.pl -i [infile] -o [outfile] -reads -unique -dist
-span -chrmap [chrmapfile] -strand -opp --silent --help
```

Parameters	
**i**	Input file. Preprocessing result file with suffix 'bedpe'
**o**	Output file. Two files are created, one with extension 'bed' and one with 'bedpe'. Both files contain all predicted splice junctions and indels.
**reads**	Minimum number of reads required in splice junction (default=2)
**unique**	Minimum number of uniquely positioned reads in splice junction (default=2)
**dist**	Maximum distance between two reads that belong to the same junction (default=20)
**span**	Only report junctions that span this distance or less (default=no limit)
**chrmap**	A tab delimited file containing a translation between chromosome names. May be required to get accurate chromosome names in the output files (see example of a chrmap file below)
**strand**	Perform strand specific analysis (default=no)
**opp**	Report junctions between opposite strands. (default=no)

NOTE! Make sure to run the program with the '-strand' parameter set if the data contains strand information for the RNAs.

Example chrmap file

The chrmap file is required when the chromosomes reported by the alignment software does not agree with the standard names. Below is an example of the chrmap file can look for the mouse mm9 assembly. In the left column are the names as reported in the SOLiD experiment and to the right are the standard names.

```
chr1       chr1
chr2       chr2
chr3       chr3
chr4       chr4
chr5       chr5
chr6       chr6
chr7       chr7
chr8       chr8
chr9       chr9
chr10      chr10
chr11      chr11
chr12      chr12
chr13      chr13
chr14      chr14
chr15      chr15
chr16      chr16
chr17      chr17
chr18      chr18
chr19      chr19
chr20      chrX
chr21      chrY
chr22      chrUn_random
```

Output files

Two output files are created, one with extension ‘bed’ and one with ‘bedpe’. Both of them contain information about all predicted splice junctions and indels but on different formats to facilitate further analysis and visualization.

BEDPE file

Here is an example of a few lines of a BEDPE result file:

```
r1chr r1start r1end   r2chr r2start r2end   name           n  s1 s2 o
chr1  5107525 5107568 chr1  5114357 5114391 event1:r18:u17 18 .  .  0
chr1  5123306 5123343 chr1  5125890 5125931 event2:r6:u5   6  .  .  0
chr1  5125977 5126018 chr1  5133831 5133868 event3:r13:u10 13 .  .  0
chr1  5140099 5140143 chr1  5152185 5152227 event4:r19:u15 19 .  .  0
chr1  6238247 6238274 chr1  6238355 6238385 event5:r2:u2   2  .  .  0
```

As seen above the file consists of 11 columns:

r1chr, r1start, r1end: The three first columns (‘r1chr’, ‘r1start’ and ‘r1end’) are the coordinates for the first part of the junction, i.e. the region covered by some split read.

r2chr, r2start, r2end: The three following columns contain the corresponding coordinates for the second part of the splice junction.

name: A unique name for each predicted splicing (or indel) on the form 'eventX:rY:uZ', where X is a unique id, Y is the total number of junction reads and Z is the number of uniquely placed junction reads.

n: total number of junction reads

s1, s2: strands for the two regions in the split alignments. In this example the SplitSeek analysis was not run using the '-strand' option and therefore dots ('.') are reported instead of '+' and '-'.

o: Number of junction reads mapping to different strands.

NOTE! A typical junction in the output file is predicted to span between 'r1end' and 'r2start'. This means that the coordinates 'r1end' and 'r2start' will correspond to the boundaries of the two exons involved in the splicing. The junction coordinates are typically predicted at very high resolution (at most 5 bp).

BED file

The BED file contains similar information as the BEDPE file but on a slightly different format. More information about the BED format can be found at http://genome.ucsc.edu.

Visualization and further analysis

BEDTools

The BEDPE file can be analyzed with the BEDTools analysis suite (Quinlan et al, *Bioinformatics*, 2010). In this way the junction coordinates can be quickly compared to various genomic features such as coordinates of genes and exons.

UCSC genome browser

The BED file can be uploaded to the UCSC genome browser (http://genome.ucsc.edu/). The figure below shows the results in an example region of the mouse genome:

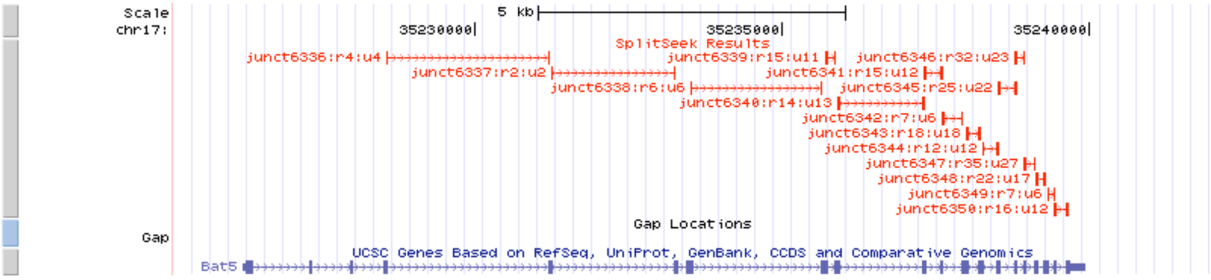

Figure 1: SplitSeek predictions (red lines) viewed in the UCSC genome browser.
